# Dodecafluoropentane Emulsion as a Radiosensitizer in Glioblastoma Multiforme

**DOI:** 10.1158/2767-9764.CRC-22-0433

**Published:** 2023-08-21

**Authors:** Jason D. Lickliter, Jeremy Ruben, Ganessan Kichenadasse, Ross Jennens, Cecelia Gzell, Ralph P. Mason, Heling Zhou, Jennifer Becker, Evan Unger, Baldassarre Stea

**Affiliations:** 1 Nucleus Network, Melbourne, Victoria, Australia.; 2 Monash University, The Alfred Hospital, Melbourne, Victoria, Australia.; 3 Flinders Centre for Innovation in Cancer, Flinders Medical Centre, Adelaide, South Australia, Australia.; 4 Epworth Healthcare, Richmond, Victoria, Australia.; 5 Genesis Care, St. Vincent's Hospital, Sydney, New South Wales, Australia.; 6 Department of Radiology, UT Southwestern, Dallas, Texas.; 7 Department of Radiology, Mayo Clinic, Scottsdale, Arizona.; 8 NuvOx Pharma, Tucson, Arizona.; 9 Department of Radiation Oncology, University of Arizona, Tucson, Arizona.

## Abstract

**Purpose:**

Glioblastoma multiforme (GBM) is a hypoxic tumor resistant to radiotherapy. The purpose of this study was to assess the safety and efficacy of a novel oxygen therapeutic, dodecafluoropentane emulsion (DDFPe), in chemoradiation treatment of GBM.

**Experimental Design:**

In this multicenter phase Ib/II dose-escalation study, patients were administered DDFPe via intravenous infusion (0.05, 0.10, or 0.17 mL/kg) while breathing supplemental oxygen prior to each 2 Gy fraction of radiotherapy (30 fractions over 6 weeks). Patients also received standard-of-care chemotherapy [temozolomide (TMZ)]. Serial MRI scans were taken to monitor disease response. Adverse events were recorded and graded. TOLD (tissue oxygenation level–dependent) contrast MRI was obtained to validate modulation of tumor hypoxia.

**Results:**

Eleven patients were enrolled. DDFPe combined with radiotherapy and TMZ was well tolerated in most patients. Two patients developed delayed grade 3 radiation necrosis during dose escalation, one each at 0.1 and 0.17 mL/kg of DDFPe. Subsequent patients were treated at the 0.1 mL/kg dose level. Kaplan–Meier analysis showed a median overall survival of 19.4 months and a median progression-free survival of 9.6 months, which compares favorably to historical controls. Among 6 patients evaluable for TOLD MRI, a statistically significant reduction in tumor T_1_ was observed after DDFPe treatment.

**Conclusions:**

This trial, although small, showed that the use of DDFPe as a radiosensitizer in patients with GBM was generally safe and may provide a survival benefit. This is also the first time than TOLD MRI has shown reversal of tumor hypoxia in a clinical trial in patients. The recommended dose for phase II evaluation is 0.1 mL/kg DDFPe.

Trial Registration: NCT02189109

**Significance:**

This study shows that DDFPe can be safely administered to patients, and it is the first-in-human study to show reversal of hypoxia in GBM as measured by TOLD MRI. This strategy is being used in a larger phase II/III trial which will hopefully show a survival benefit by adding DDFPe during the course of fractionated radiation and concurrent chemotherapy.

## Introduction

Glioblastoma multiforme (GBM) is the most common primary malignant brain tumor and has a poor prognosis. With standard therapy consisting of surgery followed by fractionated radiotherapy with concomitant and adjuvant temozolomide (TMZ), the median overall survival (OS) is only 14.6 months (1). GBM is a hypoxic tumor with median tumor partial pressure of oxygen (pO_2_) measurements approximately 1/10th the level of pO_2_ in normal brain tissue (2–6). Hypoxia in GBM tumors commonly leads to areas of necrotic tissue, a marker of hypoxia, and expression of angiogenic factors (7–9). Individual tumor pO_2_ measurements in GBM often show regions of profound hypoxia with pO_2_ measurement of <2.5 mm Hg (4).

Among the various reasons for poor outcomes in patients with GBM, tumor hypoxia is a well-known factor that is especially associated with radiotherapy resistance (10). One of the mechanisms of radiotherapy (RT) is creation of reactive oxygen species (3). It takes a 3-fold higher dose of radiation to kill hypoxic as opposed to normoxic cells (11). Because of dose-limiting toxicity (DLT), it is generally not possible to increase the dose of radiation to compensate for tumor hypoxia (12, 13). Furthermore, TMZ has been shown *in vitro* in cell culture studies of resistant glioblastoma to be more effective in a hyperoxic environment than in the setting of hypoxia (14). It is likely that hypoxia engenders the development of the chemoresistant phenotype in GBM (15–18). Accordingly, this study was performed to test a new oxygen therapeutic for its ability to reverse tumor hypoxia and potentially improve outcomes in patients with GBM undergoing chemoradiation.

A number of oxygen therapeutics have been tested in the past, including hemoglobin analogs, hyperbaric oxygen administration, and fluorocarbons (FC; ref. 3). None of these have clearly improved clinical outcomes. Prior FCs required high doses and were associated with adverse events (AE) as well as limited efficacy (19–22). Compared with the prior FCs, dodecafluoropentane (DDFP) has a lower molecular weight and boiling point (29°C). Because of these characteristics, DDFP has a superior oxygen-carrying capacity and is active at a much smaller dose (23).

DDFP emulsion (DDFPe 2% w/v) was previously studied as an ultrasound contrast agent (EchoGen; refs. 24, 25). NuvOx Pharma repurposed DDFPe (mean particle size 250 nm) for its development as an oxygen therapeutic (23, 26). In preclinical tumor models, intravenous administration of DDFPe increased oxygen levels in tumor tissue for at least 2 hours leading to an improved radiation response (27, 28). One of these studies also demonstrated that the highest probability of survival was in mice treated with DDFPe and carbogen as opposed to DDFPe while breathing air (28). In the current open-label, multicenter phase Ib/II study, we assessed the safety, tolerability, and preliminary efficacy of DDFPe as a radiosensitizer in patients with newly diagnosed GBM. This is the first report of its use in human cancer treatment.

## Materials and Methods

This open-label, multicenter phase Ib/II trial had two phases—an initial dose-exploration phase followed by a dose-expansion phase.

### Patients

Key inclusion criteria were age 18–70 years, histologically confirmed newly diagnosed GBM, no prior treatment for glioblastoma apart from surgical resection, Eastern Cooperative Oncology Group (ECOG) performance status (PS) 0–2, life expectancy of at least 3 months, either on no glucocorticoids or a stable dose for 7 days prior to enrolment, and adequate organ function. Key exclusion criteria were presence of leptomeningeal disease or multifocal glioblastoma, intracranial bleeding except for stable grade 1 hemorrhage, unstable cardiac or cerebrovascular disease, clinically significant pulmonary disease, and current anticoagulant or antiplatelet therapy except for low-dose aspirin or prophylactic doses of low molecular weight heparins. All patients provided written informed consent and the study was approved by the Institutional Ethics Committee at each site.

### Treatments

Chemoradiation consisted of 30 fractions of focal brain radiation (total 60 Gy, given as 2 Gy fractions 5 days per week for 6 weeks) with concurrent TMZ, dose of 75 mg/m^2^/day, 7 days per week for 6 weeks started 4-week postsurgical resection (29). During the chemoradiation phase, patients received oral TMZ in the morning prior to radiotherapy. Patients received DDFPe at their assigned dose level immediately prior to each fraction of radiotherapy (for up to a total of 30 doses), given as either a 30-minute intravenous infusion or a slow intravenous push lasting no less than 5 minutes. Patients breathed carbogen gas (98% oxygen and 2% CO_2_, *n* = 3) or 100% oxygen (*n* = 8) via a mask and non-rebreathing circuit during administration of DDFPe and through radiation. Supportive treatments, such as antiemetics and prophylaxis against Pneumocystis pneumonia, were given according to each institution's guidelines.

Following completion of chemoradiation, the patients had a 4-week recovery phase and then received oral TMZ at 150 (cycle 1) or 200 mg/m^2^ on days 1–5 of a 28-day cycle for a total of six cycles. Follow-up visits occurred approximately every 8 weeks from the last cycle of adjuvant TMZ for the first year and then every 3 months. Long-term follow-up continued until 12 months from the date the last patient was enrolled in the study. During each follow-up visit, patients were evaluated clinically, and results of repeat laboratory tests and a gadolinium-MRI scan were reviewed.

### DDFPe Dose Escalation

The dose-exploration phase began with an accelerated titration approach using single-patient cohorts to sequentially explore DDFPe dose levels: 0.05, 0.1, 0.17, 0.25, and 0.35 mL/kg. DLT was defined as a grade 3 or higher AE (NCI Common Terminology Criteria for Adverse Events version 4.0) that occurred within 6 weeks from commencing study therapy, deemed probably or definitely related to DDFPe and considered to be dose-limiting by the investigator. After occurrence of a DLT in 1 patient or grade 2–related AEs in 2 patients, dose escalation was required to convert to a standard 3+3 scheme to determine a MTD of DDFPe. The recommended dose (RD) for the dose-expansion phase was defined as the MTD or, if no MTD was defined, the maximum well-tolerated dose administered during dose exploration.

### Response Assessment

MRI scans with and without gadolinium contrast per standard of care were performed within 72 hours of surgery, 4 weeks after completion of chemoradiation, after cycles 3 and 6 of adjuvant TMZ and then every 3 months during follow-up. Tumor progression was assessed using Response Assessment in Neuro-oncology (RANO) criteria by the local investigator in conjunction with a local MRI radiologist (30).

### Molecular Biomarkers

Because of the effect of methylguanine-methyltransferase (MGMT) methylation status on survival and response to TMZ, and the prognostic impact of isocitrate dehydrogenase (IDH) mutations and glioma-CpG island methylator phenotype (G-CIMP), the GliomaSTRAT assay (RiboMed Biotechnologies, Inc.) was performed on archived tissue specimens on all patients (30). GliomaSTRAT interrogates both DNA methylation and mutations in the same test to determine MGMT methylation, G-CIMP profile, and IDH1 R132H mutation.

### Tissue Oxygen Level–dependent MRI

Tissue oxygen level–dependent (TOLD) contrast MRI detects hypoxia through the use of spin lattice relaxation rate (R_1_) to measure the concentration of free oxygen molecules in the interstitial fluid or plasma (i.e., pO_2_; refs. 31, 32). TOLD MRI was not available at all of the sites but was performed on patients when feasible. Baseline TOLD was performed prior to initiation of supplemental oxygen and DDFPe administration and then repeated after radiotherapy on each of days 1 and 5 during the first week of chemoradiation treatment. Four TOLD datasets were obtained per patient throughout the study using steady-state free precession Look-Locker. On day 1, baseline, two T_1_ maps were obtained, and then postradiation, four T_1_ maps were obtained. On day 5, three T_1_ maps were obtained for both timepoints. Each T_1_ map included three slice locations. Data analysis was done using MATLAB. Curve fitting was performed for every pixel to generate the T_1_ map and for region of interest for the tumor.

### Statistical Analysis

This was a phase Ib/II dose-escalation study and therefore a formal sample size calculation was not performed. The primary endpoints of the study were definition of the RD in the dose-exploration phase and progression-free survival (PFS) at 6 months (PFS-6) in the dose-expansion phase. The data cut-off date was August 31, 2017. PFS was displayed graphically and median PFS determined using the Kaplan–Meier product limit estimator. PFS was measured from the first day of chemoradiation until the first to occur out of disease progression (assessed using RANO) or death from any cause. Subjects still progression free at the end of the study were censored at the last date they were known to be alive and progression free. To estimate PFS-6 [with 90% confidence limits (CL)], the simple proportion was estimated and exact (Clopper–Pearson) 90% CLs were obtained. This approach was viable as no subjects were censored for PFS before 6 months.

OS was analyzed as described for PFS. Time to death was measured from the first day of chemoradiation therapy until the date of death from any cause. Subjects still alive at the end of the study were censored at the last date they were known to be alive. As for PFS, OS-12 could be estimated using binomial proportions and exact (Clopper–Pearson) CLs as no subjects were censored for survival prior to 12 months. The results for PFS and OS were compared descriptively with historical data for TMZ+RT alone (14.6 and 6.9 months, respectively; ref. 1). In keeping with the early phase of the study, a relaxed alpha of 10% was used.

### Data Availability Statement

The datasets generated during and/or analyzed during the current study are not publicly available due to the proprietary nature of the investigational drug product but are available from the corresponding author on reasonable request.

### Ethical Approval

The study protocol and all amendments were reviewed by the Independent Ethics Committee for each center. All procedures performed in studies involving human participants were in accordance with the ethical standards of the institutional and/or national research committee and with the 1964 Helsinki declaration and its later amendments or comparable ethical standards.

## Results

A total of 11 patients from four institutions in Australia were enrolled in the trial from July 14, 2014, to July 4, 2016. Table 1 lists patient characteristics at baseline. On the basis of review of baseline contrast enhanced MRIs obtained on day 1 of treatment, there were 2 patients with a gross total resection (GTR), while 1 patient had a biopsy only and 8 patients with subtotal resection. The mean maximal cross-sectional diameters of the residual tumors for the 9 patients who did not undergo a GTR, were 3.01 × 2.05 cm (range: 1.2–6.44 cm), while the mean largest fluid-attenuated inversion recovery (FLAIR) area encompassed 25.8 cm^2^ (range: 0–47 cm^2^). Seven of the 11 patients completed both the chemoradiation and adjuvant TMZ phases of study treatment. The remaining 4 patients were withdrawn following completion of the chemoradiation period, but prior to completion of the adjuvant TMZ period. Reasons for withdrawal were progressive disease (PD) in 2 patients and death and investigator decision in 1 patient each.

**TABLE 1:** Baseline patient characteristics

Characteristic	*N* = 11 (%)
Age – year	
Median	48
Range	30–68
Sex – no. (%)	
Male	4 (36)
Female	7 (64)
MGMT promoter status – no. (%)	
Methylated	3 (27)
Unmethylated	8 (73)
Baseline ECOG PS – no. (%)	
0	5 (46)
1	6 (54)
Extent of surgery – no. (%)	
Biopsy only	1 (9)
Gross total resection	2 (18)
Subtotal resection	8 (73)
Receiving glucocorticoids – no. (%)	
Yes	4 (36)
No	7 (64)

### Dose Escalation and Expansion

The dose-exploration phase proceeded through three single-patient cohorts at sequential doses of 0.05, 0.1, and 0.17 mL/kg. No protocol defined DLT was observed during the 6 weeks after commencing study therapy. However, 2 patients developed histopathologically confirmed grade 3 radiation necrosis at later timepoints. This occurred at each of the 0.1 and 0.17 mL/kg dose levels, leading to surgical resections at 9.4 and 1.8 months from starting study treatment, respectively. Identification of these two cases of grade 3 radiation necrosis (attributed as possibly related to DDFPe) resulted in termination of dose escalation and the enrolment of an additional 5 patients at the 0.1 mL/kg dose level. Because no DLTs or further events of radiation necrosis were observed, 0.1 mL/kg was declared the RD and this dose level was expanded by an additional 3 patients.

### Safety

Table 2 shows all-cause grade 2 and higher AEs in the 11 patients enrolled in the trial. Ten patients (91%) experienced at least one AE of grade 2–4. Among these, the most common AEs were fatigue, neutrophil count decreased, platelet count decreased, and nausea. Three patients had study therapy interrupted during chemoradiation due to an AE, specifically due to either decreased platelet count or atypical pneumonia. No acute toxicity due to intravenous infusions of DDFPe was observed at any dose level.

**TABLE 2:** All-cause AEs of grade 2 or greater severity

Adverse event	Grade 2	Grade 3	Grade 4	Total
Fatigue	5 (46%)	1 (9%)	—	6 (55%)
Neutrophil count decreased	2 (18%)	1 (9%)	1 (9%)	4 (36%)
Platelet count decreased	—	1 (9%)	3 (27%)	4 (36%)
Nausea	3 (27%)	—	—	3 (27%)
Deep vein thrombosis	2 (18%)	—	—	2 (18%)
Pyrexia	—	2 (18%)	—	2 (18%)
Radiation necrosis	—	2 (18%)	—	2 (18%)
Alopecia	1 (9%)	—	—	1 (9%)
Anemia	—	1 (9%)	—	1 (9%)
Atypical pneumonia	1 (9%)	—	—	1 (9%)
Back pain	1 (9%)	—	—	1 (9%)
Catheter site discharge	1 (9%)	—	—	1 (9%)
Conjunctivitis	1 (9%)	—	—	1 (9%)
Constipation	1 (9%)	—	—	1 (9%)
Cushingoid	1 (9%)	—	—	1 (9%)
Decreased appetite	1 (9%)	—	—	1 (9%)
Drug-induced liver injury	—	1 (9%)	—	1 (9%)
Head injury	1 (9%)	—	—	1 (9%)
Headache	1 (9%)	—	—	1 (9%)
Hypoalbuminemia	—	1 (9%)	—	1 (9%)
Hypokinesia	—	1 (9%)	—	1 (9%)
Lymphocyte count decreased	—	—	1 (9%)	1 (9%)
Myopathy	1 (9%)	—	—	1 (9%)
Neck pain	1 (9%)	—	—	1 (9%)
Pain in extremity	1 (9%)	—	—	1 (9%)
Respiratory tract infection	1 (9%)	—	—	1 (9%)
Subclavian vein thrombosis	1 (9%)	—	—	1 (9%)
Syncope	—	1 (9%)	—	1 (9%)
Vertigo	1 (9%)	—	—	1 (9%)
Vomiting	1 (9%)	—	—	1 (9%)
White blood cell count decreased	—	—	1 (9%)	1 (9%)

NOTE: Table entries are the number (%) of patients with the corresponding AE. Only events of ≥ grade 2 are shown. Each AE is counted only once in each affected patient, with only the worst grade experienced indicated. Multiple different AEs in a single patient are shown as separate AEs in different rows.

Two patients experienced AEs of grade 3 radiation necrosis after chemoradiation and DDFPe. Patient 01-003 was a 48-year-old female with a partially resected left parietal glioblastoma who received DDFPe at 0.17 mL/kg. She underwent a repeat resection 54 days from the start of chemoradiation after presenting with headaches, increased right hemiparesis, and a new MRI finding of thick enhancing margins surrounding the original surgical cavity. Postoperatively, her headache resolved and the hemiparesis improved. Histopathology showed extensive radiation necrosis as the predominant finding with some viable tumor tissue also present. Patient 01-002 was a 65-year-old female with a partially resected temporooccipital glioblastoma who received DDFPe 0.1 mL/kg during chemoradiation and subsequently completed six cycles of adjuvant TMZ. She was admitted to the hospital 7 weeks after the final TMZ cycle with severe headache and vomiting and an MRI scan showed an increase in enhancing tissue surrounding the surgical cavity. A repeat resection was performed, and histopathology showed extensive radiation necrosis and recurrent GBM.

### Efficacy

**FIGURE 1:**
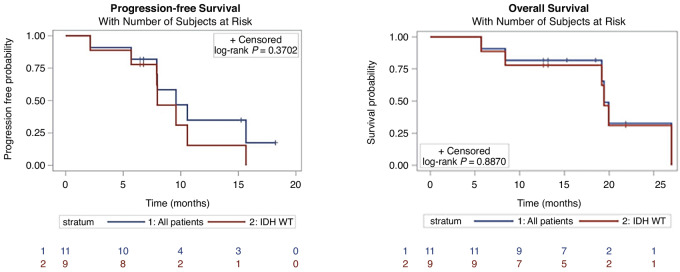
Kaplan–Meier analysis of PFS (left) and OS (right) for all 11 patients and for the 9 patients with IDHwt.

Median follow-up from the start of chemoradiation was 18.5 months (range: 5.7–27.0). Kaplan–Meier plots of PFS and OS are shown in Fig. 1. Median PFS was 9.6 months (90% CLs, 7.9–15.7), which suggests an improvement over the historical value of 6.9 months as the lower CL is > 6.9. PFS-6 was 82% (exact 90% CL, 53.0–96.7). For the subgroup of patients with an unmethylated MGMT promoter (*n* = 8), the median PFS was 8.0 months (90% CL, 5.7–10.6) and PFS-6 was 75% (exact 90% CL, 40.0–95.4). There were six deaths during the study with none related to study treatment. The median OS was 19.4 months (90% CL, 19.2–undefined). This suggests a survival benefit compared with the historical value of 14.6 as the lower CL is > 14.6. OS at 6 and 12 months was 91% (exact 90% CL, 63.6–99.5) and 82% (exact 90% CL, 53.0–96.7), respectively. For the subgroup of patients with an unmethylated MGMT promoter (*n* = 8), median OS was 19.3 months (exact 90% CL, 8.4–19.9).

Furthermore, excluding the 2 patients with the IDH mutations from the survival analysis made no difference to the median OS, which is still 19.4 months (CL, 8.8–undefined) likely because those 2 patients were alive at the end of the study, and hence are censored in the analysis and their follow-up time is <19 months. However, a difference was noted in the PFS, with the 9 patients harboring IDH-wildtype (IDHwt) having a lower median PFS = 7.95 months (CL, 5.7–10.6) compared with the whole group of 11 patient with a median PFS of 9.6 months (see below).

**FIGURE 2:**
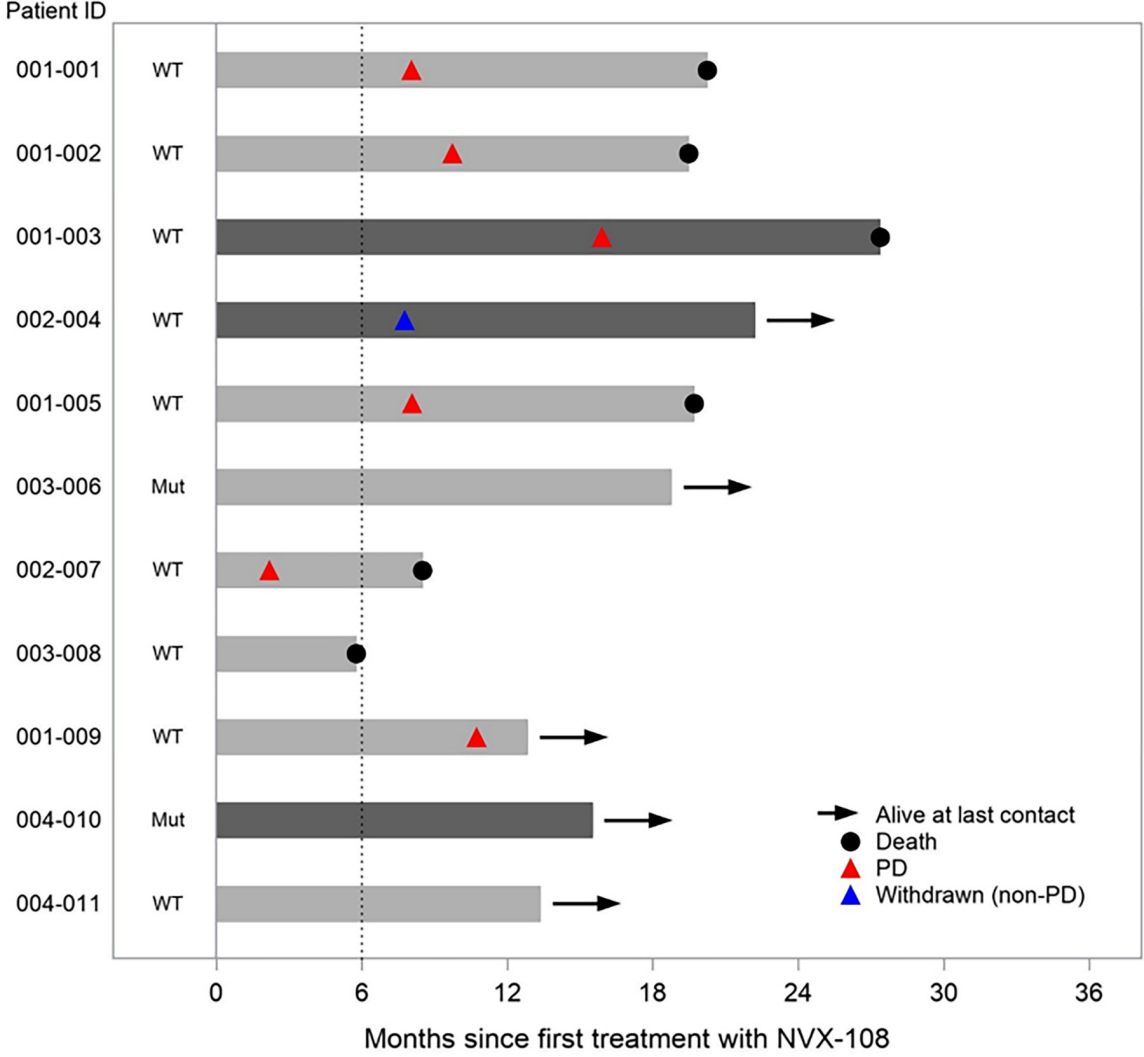
Swimmer plot for patients with GBM treated with DDFPe, radiotherapy, and TMZ, showing PD and survival in relation to tumor MGMT promoter methylation status (methylated indicated by dark gray bars and unmethylated by light gray bars) and IDH1 mutation status. WT, wild type IDH1; Mut, R132H IDH1 mutation present; PD, progressive disease.

The swimmer plot in Fig. 2 summarizes key endpoints including PD (per RANO criteria), withdrawal from the study for reasons other than PD and survival at last follow-up, shown in relation to the predictive biomarkers of tumor MGMT promoter methylation and IDH1 mutation status. Eight of the total 11 patients with GBM had an unmethylated MGMT promoter and IDH was mutated in 2 patients and wild type in 9 patients. Patient 03-008 died at 5 months after beginning study treatment, without evidence of PD. Patients 003-006 and 004-010 (both with IDH mutations) were alive and progression free at 18.5 and 15.3 months, respectively, after starting study treatment. Patient 002-004 was withdrawn from the study at 7.7 months per decision of the Investigator.

### Tissue Oxygenation Biomarker

**FIGURE 3:**
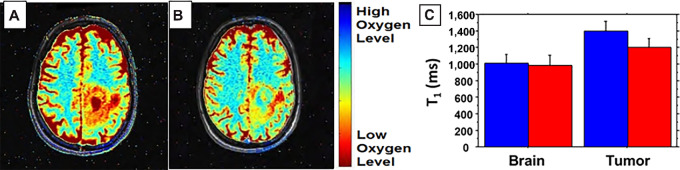
TOLD MRI of patient receiving 0.17 mL/kg DDFPe pretreatment (**A**) and posttreatment (**B**) shows reversal of tumor hypoxia. T1 of tumor (mean ± SD) was 1,454 ± 75 ms preadministration of DDFPe and decreased to 1,281 ± 77 ms after treatment. NMR relaxation measurements before and after infusion of DDFPe (**C**). NMR T1 measurements represent mean ± SD for the 4 patients encompassing tumor or contralateral normal brain imaged at 1.5 T. Baseline measurements (blue) were performed before administration of DDFPe, and postmeasurements (red) were made following intravenous administration of DDFPe, while patients breathed oxygen or carbogen following irradiation. Normal brain tissue showed no significant response (*P* = 0.70), while tumor showed significant change following administration of DDFPe and irradiation (*P* = 0.0059). Considering all measurements, tumor tissues had significantly longer T1 relaxation times than normal brain (*P* < 0.0001). At baseline brain T1 was significantly shorter than tumor (*P* < 0.0001) and this remained so after administering DDFPe (*P* = 0.0045).

Patients underwent TOLD MRI, an oxygen-sensitive imaging technique, before and after treatment with DDFPe, supplemental oxygen and radiation on both days 1 and 5 of their first week of treatment (Fig. 3).

A total of 6 patients were included in the full image analysis set as not all sites were equipped to perform the imaging. Of the 6 patients, 4 patients were imaged on a 1.5T scanner and 2 patients on a 3T scanner. Median time for posttreatment TOLD imaging was 113 minutes (range: 35–204 minutes) after completion of DDFPe dose administration. There was a significant decrease in T_1_ values from pretreatment to posttreatment for the tumor tissue, but not in normal tissue, indicating improved tumor oxygenation after DDFPe treatment (Fig. 3C).

## Discussion

We studied DDFPe given as an intravenous infusion prior to each fraction of radiotherapy when added to a standard chemoradiation regimen in patients with newly diagnosed GBM. The combination treatment was generally well tolerated and AEs were largely as expected after chemoradiation alone in this patient group. However, symptomatic grade 3 radiation necrosis leading to surgical resection was observed in 2 of the initial 3 patients receiving DDFPe with chemoradiation (one each at the 0.1 and 0.17 mL/kg dose levels). This resulted in termination of dose escalation and expansion of the 0.1 mL/kg dose level, with no further events of radiation necrosis observed in an additional 8 patients. It is noteworthy that a recent large, randomized trial utilizing radiation and TMZ in patients with newly diagnosed GBM did not report any serious radiation necrosis AEs, including in the 309 patients who received chemoradiation alone (33). The patient receiving 0.17 mL/kg of DDFPe in the current study developed radiation necrosis earlier than the single affected patient at 0.1 mL/kg (1.8 vs. 9.4 months after starting treatment, respectively). However, because only 1 patient was treated at 0.17 mL/kg, no definitive conclusions about the effect of DDFPe dose on the incidence and severity of radiation necrosis after radiotherapy and TMZ can be made.

With a median PFS of 9.6 months, PFS-6 of 82% and median OS of 19.4 months, the efficacy of combined DDFPe, radiotherapy, and TMZ observed in our trial compares favorably to published results of chemoradiation alone from large phase III trials (29, 34), suggesting an improvement in both PFS and OS. Because of trial design, we could not follow the patients indefinitely; at last follow-up 5 of 11 patients were still alive, so the OS is likely > 19.4 months. Nonetheless, the number of patients in our study was small and the confidence intervals around these efficacy variables are wide.

TOLD MRI scans showed significant decreases in T_1_ of tumor tissue after DDFPe consistent with reversal of tumor hypoxia, suggesting that the duration of action is at least 1–2 hours following administration of DDFPe (Fig. 3). There was no significant change in oxygenation of normal brain tissue, which indicates the study drug does not have a potentially harmful hyperoxygenating effect on normal tissue (Fig. 3C). The results are encouraging, but additional studies are required in which the baseline TOLD scan includes supplemental oxygen to then quantify the oxygenating effects of the study drug only in the post-TOLD scan.

TOLD MRI is a novel technique that has potential as a non-invasive prognostic imaging biomarker in terms of assessing tissue oxygenation in hypoxic tumors (34). Hypoxia is not identified or quantified in current clinical oncology practice (35). TOLD MRI has been applied to investigations of brain tumors in rats and mice (36, 37) and to human brain tumors to characterize response to an oxygen gas breathing challenge (38). The current study is the first in-human use of TOLD MRI to monitor the efficacy of an oxygen therapeutic to reverse hypoxia in GBM as part of a therapeutic clinical trial. Additional human studies are needed to confirm the reproducibility of the biomarker. Changes in tumor hypoxia accompanying administration of the mitochondrial inhibitor atovaquone in patients with non–small cell lung cancer were observed using PET based on ^18^F-misonidazole (39), but TOLD avoids the logistical challenges of PET.

The active substance of the emulsion, DDFP, is cleared via exhalation. As normal human body temperature is 37°C, DDFP is cleared much faster compared with previously tested FCs due to its lower boiling point of 29°C. In blood, the elimination half-life of DDFP is biexponential, with an initial half-life of 1.8–2.5 minutes for doses of 0.01–0.1 mL/kg, and a terminal half-life of about 90 minutes in humans, and the recovery of DDFP in expired air at 120 minutes ranges from 95% to 103% (average 98% ± 19%) over the same dose range (40). Fluosol (20% perfluorodecalin emulsion) is the only other FC to have been tested in clinical trials to reverse tumor hypoxia in glioma (21, 22). However, the large doses of Fluosol required, led to significant adverse events. On a gram weight basis of FC, the doses of DDFP used in this study were at least 800 times lower than the dose of FC used in Fluosol (3). DDFPe is the first FC emulsion with sufficient safety margin to enable administration during each fraction of radiotherapy.

## Conclusion

Repeat administration of DDFPe prior to each fraction of radiotherapy was feasible and tolerated in patients with newly diagnosed GBM undergoing chemoradiation treatment. Dose escalation was terminated because of the development of symptomatic radiation necrosis in 2 patients that was considered possibly related to study treatment drug and the RD of DDFPe for this schedule and patient group was determined to be 0.10 mL/kg. TOLD MRI showed selective decreases in T_1_ of tumor tissue consistent with tumor reoxygenation. This is the first time that TOLD MRI has shown reversal of tumor hypoxia in patients with an oxygen therapeutic. PFS and OS were increased compared with historical controls, but our small sample size limits any firm conclusions in this regard. Given these results, we believe that further study of DDFPe as a chemotherapy and radiation sensitizer is warranted, and the FDA has allowed a phase II randomized, prospective trial of DDFPe in combination with chemoradiation treatment of primary GBM.
